# A Reliability-Based Method to Sensor Data Fusion

**DOI:** 10.3390/s17071575

**Published:** 2017-07-05

**Authors:** Wen Jiang, Miaoyan Zhuang, Chunhe Xie

**Affiliations:** School of Electronics and Information, Northwestern Polytechnical University, Xi’an 710072, China; zhuang-my@mail.nwpu.edu.cn (M.Z.); xiechunhe@mail.nwpu.edu.cn (C.X.)

**Keywords:** sensor data fusion, Dempster–Shafer evidence theory, Gaussian distribution, reliability-based BBA

## Abstract

Multi-sensor data fusion technology based on Dempster–Shafer evidence theory is widely applied in many fields. However, how to determine basic belief assignment (BBA) is still an open issue. The existing BBA methods pay more attention to the uncertainty of information, but do not simultaneously consider the reliability of information sources. Real-world information is not only uncertain, but also partially reliable. Thus, uncertainty and partial reliability are strongly associated with each other. To take into account this fact, a new method to represent BBAs along with their associated reliabilities is proposed in this paper, which is named reliability-based BBA. Several examples are carried out to show the validity of the proposed method.

## 1. Introduction

In practical applications, there are various interferences in the working environment. Sensor data fusion technology can combine the related information from multiple sensors to enhance the robustness and safety of a system [1,2]. Hence, this technique has received significant attention in many fields, such as target tracking and recognition [3,4], complex network [5,6,7] and image processing [8,9,10]. Besides, information gathered from sensors is usually uncertain due to the change of the environment. It can degrade the performance of the information fusion system. Thus, how to handle uncertain information is a vital issue in the sensor fusion system. To solve this problem, many theories are presented by domestic and foreign scholars. Fuzzy sets theory was first introduced by Zadeh [11] in 1965 as an extension of the classical notion of set. It can be used in a wide range of domains in which information is incomplete or imprecise [12,13,14]. Dempster–Shafer evidence theory (evidence theory) acts as the pioneer in data fusion algorithms, which was proposed by Dempster [15] and extended by Shafer [16] subsequently. It is capable of managing epistemic and aleatoric uncertainty due to its framework. Possibility theory was introduced in 1978 by Zadeh [17]. It describes reasonably the meaning of information, especially the meaning of incomplete information within a possibilistic framework [18,19,20,21], which could be seen as the theory interconnecting fuzzy sets and evidence theory.

Within these theories, evidence theory has a good performance to process the uncertain information without the prior probability, which contributes to its wide application [22,23]. However, counter-intuitive results may be obtained when dealing with highly conflicting evidence. A famous example was illustrated by Zadeh [24]. Since then, many methods have been proposed to address this issue [25,26,27]. Martin et al. [28] proposed a conflict measures of a group of experts based on the distance of basic belief assignments. Smarandache et al. [29] presented a new normalization of a measure called contradiction to characterize the degree of discord or conflict inside a body of evidence. Martin [30] defined a conflict measure to quantify how the focal elements of two mass functions are included together. Deng et al. [31] considered a biological and evolutionary perspective to study the combination of evidences. Another open issue in evidence theory is how to determine the basic belief assignment (BBA). So far, many methods to generate BBA have been put forward. Denoeux [32] determined BBA by minimizing the mean squared differences between the classifier outputs and target values. Xu et al. [33] calculated an interval BBA from this matching degree by the modified Latin hypercube sampling Monte Carlo technique. Tabassian et al. [34] determined BBA in which the class memberships of training data are subject to ambiguity. A BBA method based on probability families encoded by possibility distributions and belief functions is presented by Baudrit and Dubois [35]. Mönks et al. [36,37] defined a fuzzy basic belief assignment (μBBA) based on α-cut of fuzzy membership.

Comparing with the μBBA, the idea of the proposed BBA determination method is similar to the μBBA method to some extent. We both use the fuzzy membership function to obtain the degree of membership to the respective propositions, and the degree of membership is applied as BBA. However, the proposed method is still very different from the μBBA method in some aspects. First, the BBA of the μBBA method is defined in the real line. Namely, the determination of BBA is the determination of the possibility of sensor signal belonging to each interval proposition, which is the determination of value ranges. The BBA of the proposed method is defined in categorical data. Namely, the determination of the sensor signal is the determination of the category. Besides, in the proposed method, the fuzzy membership function is used to model the feature of each category proposition. However, in the μBBA method, the fuzzy membership function is used to represent the knowledge of possibility, and the proposition is modeled by an interval. Hence, the proposed method and the μBBA method are different. They are applicable to the different application backgrounds.

In reality, information is often not just uncertain, but also partially reliable. If we only consider one of them, then the whole complexity of real-world information cannot completely be covered, which may cause the incorrect fusion results. Hence, reliability evaluation is indispensable in practical applications [38,39]. Guo et al. [40] presented a new framework for sensor reliability evaluation in classification problems based on evidence theory. Yuan et al. [41] took the static reliability and dynamic reliability into consideration to handle the conflicting evidence. However, the reliability of these methods is measured from the support degree (consistency) among BBA. Namely, the reliability is obtained based on the given BBA, rather than from the information sources, which may lose part of the source information. Glock et al. [42] used the concepts of majority observation and consistency to monitor the sensor reliability based on the possibilistic framework. Ehlenbröker et al. [43] proposed a method to generate a consistency-based reliability assessment for sensors, which is utilized to detect sensor defects based on groups of sensors instead combining all sensors at once. In the two reliability methods, the whole measure of reliability is time dependent. Besides, the static reliability is a prior reliability based on expert knowledge, and the dynamic reliability considers its former reliability and the consistency of observations. Comparing with that, the measure of reliability is not time dependent in this paper. The reliability is obtained based on the measure of sensor capability to distinguish the different targets. The capability to discriminate the difference classes is large under a certain attribute, and the reliability of the generated BBA is larger under this attribute.

The existing BBA methods pay more attention to the uncertainty of information, but do not simultaneously consider the reliability of information sources. In fuzzy sets theory, Zadeh [44] proposed the concept of the Z-number in 2011, which is an ordered pair of fuzzy numbers denoted by Z=(A,B). The first component *A* is a fuzzy measurement of the uncertainty. The second component *B* is a measurement of the reliability of A. The Z-number can simultaneously describe the reliability of information sources. Originating from the idea of the Z-number, a new method to represent BBAs along with their associated reliability is proposed in this paper, which is named reliability-based BBA. Reliability-based BBA (BBA,R) is an ordered pair; its first component BBA is a mass function, and the second component *R* is a measurement of the reliability of the first component BBA.

The rest of this paper is organized as follows. The relevant concepts of evidence theory and pignistic probability are briefly recalled in Section 2. In Section 3, a reliability-based BBA is proposed. In Section 4, this method is compared with other methods by several examples. The conclusion is presented in Section 5.

## 2. Preliminaries

In this section, the relevant concepts of evidence theory and pignistic probability are briefly recalled.

### 2.1. Dempster–Shafer Evidence Theory

Evidence theory was introduced by Dempster [15] and then developed by Shafer [16]. It includes the following concepts: frame of discernment, mass function and Dempster’s combination rule, etc. These concepts contribute to its good performance in handling the uncertainty information [45,46,47].

#### 2.1.1. Frame of Discernment

Let Θ be a set of *N* mutually-exclusive and collectively-exhaustive hypotheses, defined as:

(1) $$\Theta =\{\theta_{1},\theta_{2},\cdots ,\theta_{i},\cdots ,\theta_{N}\}$$

where Θ is called a frame of discernment. The power set of Θ is composed with $2^{\Theta }$, namely:

(2) $$2^{\Theta }=\left\{\emptyset ,\left\{\theta_{1}\right\},\cdots \left\{\theta_{N}\right\},\left\{\theta_{1},\theta_{2}\right\},\cdots ,\left\{\theta_{1},\theta_{2},\cdots \theta_{i}\right\},\cdots ,\Theta \right\}$$

where ∅ is denoted as the empty set. The N subsets containing only one element each are called the singleton subset proposition; the subsets containing more than one element each are called the compound subset proposition.

#### 2.1.2. Mass Function

A mass function *m* is a mapping from $2^{\Theta }$ to [0,1], formally defined as:

(3) $$m:2^{\Theta }\to \left[0,1\right],$$

which satisfies the following conditions [16]:

(4) $$\left\{\begin{array}{c} \sum_{A\subset 2^{\Theta }}m\left(A\right)=1\\ m(\emptyset )=0. \end{array}\right.$$

The mass function *m* is also called the BBA function. Any subset A of $2^{\Theta }$, such that m(A)>0, is called a focal element.

#### 2.1.3. Dempster’s Combination Rule

Suppose $m_{1}$ and $m_{2}$ are two mass functions in the same frame of discernment. Dempster’s combination rule, which is denoted as $m=m_{1}⊕m_{2}$, is defined as follows [15]:

(5) $$m\left(A\right)=\left\{\begin{array}{cc} \frac{\sum_{B\cap C=A}m_{1}\left(B\right)m_{2}\left(C\right)}{1-k}, & A\neq \emptyset \\ 0, & A=\emptyset \end{array}\right.$$

where:

(6) $$k=\sum_{B\cap C=\emptyset }m_{1}\left(B\right)m_{2}\left(C\right).$$

Here, *k* is regarded as a measure of conflict between $m_{1}$ and $m_{2}$. The value of *k* is larger, and the conflict between the evidence is larger.

#### 2.1.4. Discounting

Assuming that a BBA has a support degree of α, where 0≤α≤1, then this BBA is discounted by the following discounting rule [16]:

(7) $$\left\{\begin{array}{c} m^{\alpha }\left(A\right)=\alpha \times m\left(A\right)\;\;\;\forall A\subset 2^{\Theta }\\ m^{\alpha }\left(\Theta \right)=\alpha \times m\left(\Theta \right)+\left(1-\alpha \right) \end{array}\right.$$

where *A* is any subset of the power set of the frame of discernment Θ.

### 2.2. Pignistic Probability

The pignistic probability function is introduced by Smets and Kennes [48] for decision making. Its procedure corresponds to the insufficient reason principle: if you need to build a probability distribution on *n* elements, given a lack of information, give a probability 1/n to each element. This procedure is repeated for each mass *m*. Let BetP be the pignistic probability distribution so derived. For all propositions A∈Θ,

(8) $$BetP\left(A\right)=\sum_{A\subseteq B\subseteq 2^{\Theta }}\frac{1}{\left|B\right|}\cdot \frac{m(B)}{1-m(\emptyset )}$$

where ∅ is denoted as the empty set. *B* is the proposition in mass function *m*, and |B| is the cardinality of *B*.

## 3. The Proposed Method

**Figure 1:**
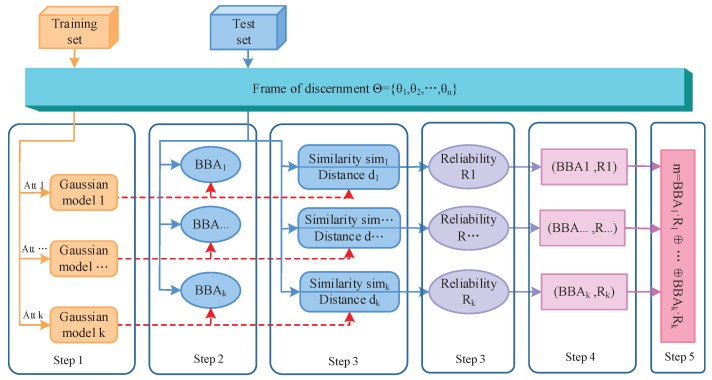
Flowchart of the proposed method.

In this section, the method of generating reliability-based BBA is given in detail. As shown in Figure 1, the method is expounded from five parts. In the first part, the models of training samples are built using the Gaussian membership functions. In the second part, according to the matching degree between the test sample and the attribute model, the first component BBA is generated, which is based on a previous work in the literature [49]. In the third part, the second component *R* (reliability of BBA) is measured, where both the similarity among classes under a certain attribute (static state) and the distance between the test sample and the models (dynamic state) are taken into account. The main contribution of this paper focuses on this part. Then, a reliability-based BBA (BBA,R) is obtained. Each (BBA,R) is discounted by the obtained reliability. Finally, these reliability-based BBAs are fused based on Dempster’s combination rule. At the stage of modeling of sensor information, the uncertainty and the reliability of the information source are simultaneously considered in the proposed method, which can obtain a more adequate construction for the description of real-world information.

### 3.1. The Modeling of Each Attribute

Due to the change of environment, the sensor data have usually a certain degree of fuzziness. In this case, the membership function can be used to represent the sample feature. Besides, there are some interruptions in the working process of the sensor, such as the mechanical noise and electromagnetic waves. In this case, the probability density function of the measured value of the same physical quantity is generally regarded as a form of Gaussian distribution. The Gaussian distribution possesses the following advantages [50]: first, if the error can be seen as the superposition of many independent random variables, then the error is supposed to have the form of the Gaussian distribution based on the central limit theorem. Second, many of the probability distributions of random variables in production and scientific experiments can be approximately described by the Gaussian distribution. Hence, the modeling of the training samples is built based on the Gaussian membership function in this paper.

Assuming that *X* is a sample space of the training set, then the Gaussian membership function of each attribute is defined as follows:

μ(x):X→[0,1],x∈X.

μ(x) can be gained by the following steps:

1. Suppose that there are *n* classes, namely the frame of discernment $\Theta =\{\theta_{1},\theta_{2},\cdots ,\theta_{n}\}$. Each class $\theta_{i}\;\left(i=1,2,\cdots ,n\right)$ has *k* attributes.
2. For the training samples of class $\theta_{i}$ in the *j*-th attribute, the mean value ${\bar{X}}_{ij}$ and the standard deviation $\sigma_{ij}$ are calculated respectively as follows:
  (9) $$\begin{array}{c} {\bar{X}}_{ij}=\frac{1}{N}\sum_{l=1}^{N}x_{ijl},\\ \sigma_{ij}=\sqrt{\frac{1}{N-1}\sum_{l=1}^{N}{\left(x_{ijl}-{\bar{X}}_{ij}\right)}^{2}}, \end{array}$$
  where i=1,2,⋯,n and j=1,2,⋯,k. *N* is the training sample size of class $\theta_{i}$. $x_{ijl}$ is the attribute value of the *j*-th attribute from the *l*-th training sample in class $\theta_{i}$.
3. The Gaussian membership function of the *j*-th attribute of class $\theta_{i}$ is generated as follows:
  (10) $$\mu_{i}^{j}\left(x\right)=exp\left[-\frac{{\left(x-{\bar{X}}_{ij}\right)}^{2}}{2\sigma_{ij}^{2}}\right]$$
  where $-3\sigma_{ij}\leq x\leq 3\sigma_{ij}$, i=1,2,⋯,n and j=1,2,⋯,k.

### 3.2. Reliability-Based BBA Generation Method

In this section, a reliability-based BBA is introduced from two parts: one is the determination of BBA; another is the reliability measurement of BBA.

#### 3.2.1. The Determination of BBA

**Figure 2:**
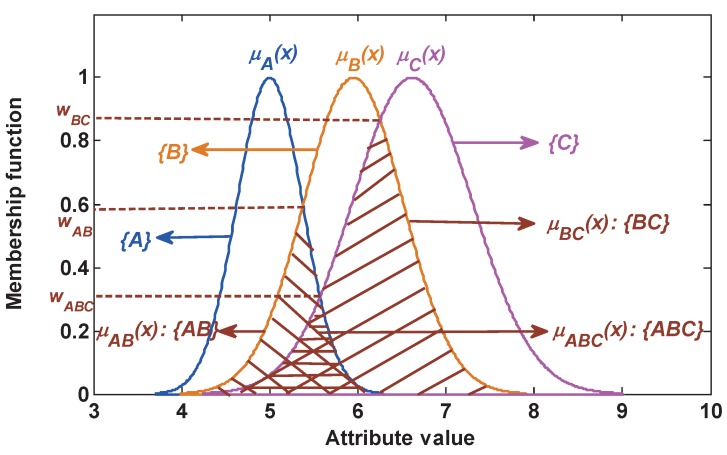
The modeling of the singleton subset and compound subset.

In this section, a nested structure BBA function is introduced. As described in Figure 2, a singleton subset proposition is modeled by a Gaussian membership function. For example, the singleton subset proposition {A} is produced by the membership function $\mu_{A}\left(x\right)$. The proposition with two elements is represented by the intersection of two singleton subset propositions. For instance, the compound subset proposition with two elements {AB} can be constructed as follows:

(11) $$\mu_{AB}\left(x\right)=min\left(\mu_{A}\left(x\right),\mu_{B}\left(x\right)\right).$$

Namely, {AB} is defined by the intersection $\mu_{AB}\left(x\right)$ of {A} with {B}. Further, a compound subset proposition with three elements can be constructed by the intersection among three singleton subset propositions. For example, {ABC} can be constructed as follows:

(12) $$\mu_{ABC}\left(x\right)=min\left(\mu_{A}\left(x\right),\mu_{B}\left(x\right),\mu_{C}\left(x\right)\right).$$

Suppose that $G(G\subset 2^{\Theta })$ is a proposition and *t* is the feature information of a test sample under a certain attribute. The matching degree between *t* and *G* implies the plausibility of this test sample belonging to this proposition, which is defined as follows:

(13) $$Pl\left(G|t\right)=\mu_{G}\left(x\right){|}_{x=t}$$

where *G* can be a singleton subset proposition or a compound subset proposition. Equation (13) indicates that the plausibility is determined by the intersection between functions $\mu_{G}\left(x\right)$ and x=t.

That is, the plausibility of a test sample belonging to these propositions {A}, {AB} and {ABC} is denoted respectively as follows:

(14) $$\begin{array}{c} Pl\left(A|t\right)=\mu_{A}\left(x\right){|}_{x=t},\\ Pl\left(AB|t\right)=\mu_{AB}\left(x\right){|}_{x=t},\\ Pl\left(ABC|t\right)=\mu_{ABC}\left(x\right){|}_{x=t}. \end{array}$$

**Figure 3:**
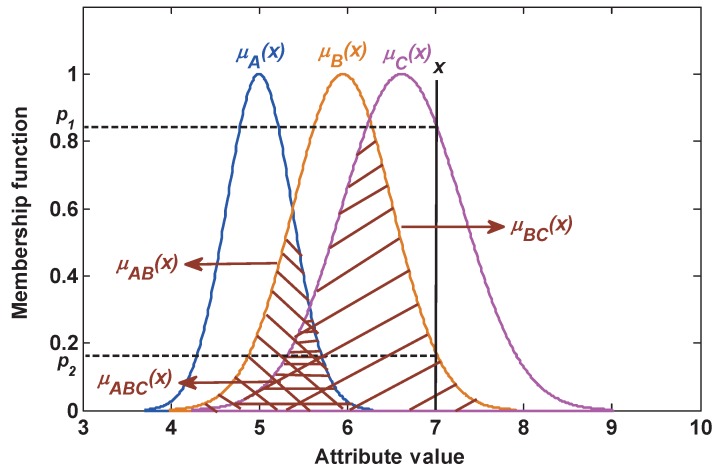
The determination of BBA.

Then, the plausibility function, which measures the matching degree between the test sample and class proposition, is used to determinate BBA in this paper. Note that when the plausibility of a singleton subset proposition is equal to the plausibility of a compound subset proposition, this plausibility is only assigned to the compound subset proposition as its BBA. For example, as shown in Figure 3, for the test sample *x*, the plausibility of this test sample belonging to each proposition can be given as:

$$\begin{array}{c} Pl\left(B|t\right)=\mu_{B}\left(x\right){|}_{x=7}=p_{2},\\ Pl\left(C|t\right)=\mu_{C}\left(x\right){|}_{x=7}=p_{1},\\ Pl\left(BC|t\right)=\mu_{BC}\left(x\right){|}_{x=7}=p_{2},\\ Pl(A|t)=Pl(AB|t)=Pl(AC|t)=Pl(ABC|t)=0 \end{array}$$

Then, the BBA of each proposition is obtained as follows:

$$\begin{array}{c} m\left(C\right)=Pl\left(C|t\right)=p_{1},\\ m\left(BC\right)=Pl\left(BC|t\right)=p_{2},\\ m(A)=m(B)=m(AB)=m(AC)=m(ABC)=0 \end{array}$$

where since $Pl\left(B|t\right)=Pl\left(BC|t\right)=p_{2}$, the plausibility $p_{2}$ is assigned to the compound subset proposition BC, namely $m\left(BC\right)=p_{2}$, but m(B)=0.

Considering that the cumulative sum of the above gained BBAs may not be equal to one, the following rules are given to normalize BBAs: if the total sum is greater than one, these BBAs are normalized; if the total sum is less than one, the redundancy belief value 1-∑BBA is assigned to the universal set Θ, namely that is assigned to the unknown.

#### 3.2.2. The Measurement of the Reliability of BBA

**Figure 4:**
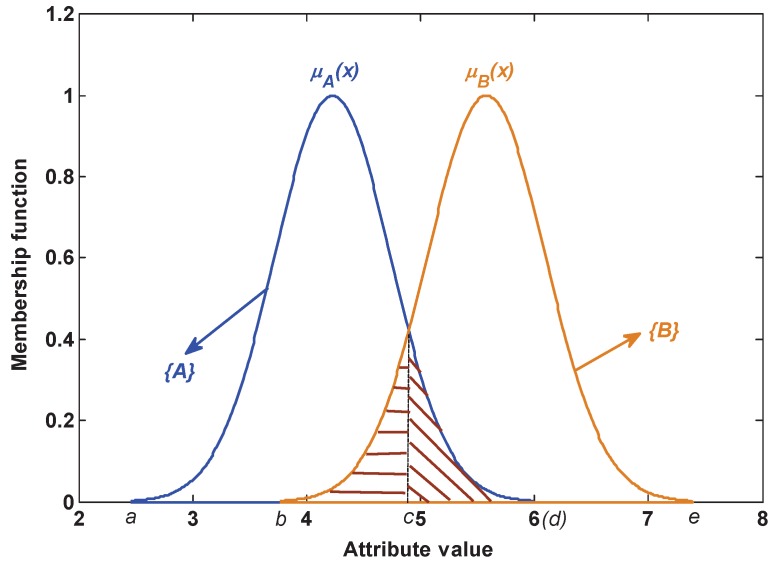
The overlapping area of the static attribute model.

As shown in Figure 4, there exist two kinds of potential possibility of false classification: one is that class *A* is incorrectly recognized as class *B*, which is defined as P(B∣A); the other is that class *B* is incorrectly recognized as class *A*, which is defined as P(A∣B). The potential possibility leads to the generated BBA being not wholly reliable. This issue is not considered in the existing BBA methods. To address this issue, a method of measuring the BBA reliability is proposed in this paper.

1. The static reliability index based on the attribute model:

Assume there are two kinds of class *A* and *B*; from Figure 4, we can know that if a test sample comes from class *A* and its value locates in the range from *c* to *d*, the possibility of the false BBA $P\left(B|A\right)=\int_{c}^{d}\mu_{A}\left(x\right)dx/\int_{a}^{d}\mu_{A}\left(x\right)dx$. If a test sample comes from class *B* and its value locates in the range from *b* to *c*, the possibility of the false BBA $P\left(A|B\right)=\int_{b}^{c}\mu_{B}\left(x\right)dx/\int_{b}^{e}\mu_{B}\left(x\right)dx$. Hence, the total error rate of BBA is P=P(B∣A)+P(A∣B). Namely, under a certain model ($\int_{a}^{d}\mu_{A}\left(x\right)dx$ and $\int_{b}^{e}\mu_{B}\left(x\right)dx$ are invariable), if the overlapping area $\int_{c}^{d}\mu_{A}\left(x\right)dx$ or $\int_{b}^{c}\mu_{B}\left(x\right)dx$ is larger, the similarity among classes is larger, then the possibility of generating the false BBA is larger, and vice versa. As analyzed above, a method of measuring the reliability of BBA is proposed based on the similarity among the attribute model, which is detailed as follows.

In the attribute j(j=1,2,⋯,k), the similarity between Classes 1 and 2 is defined as:

(15) $$sim_{12}^{j}=sim\left(\mu_{1}^{j}\left(x\right),\mu_{2}^{j}\left(x\right)\right)=\frac{\int \mu_{12}^{j}\left(x\right)dx}{\int \mu_{1}^{j}\left(x\right)dx+\int \mu_{2}^{j}\left(x\right)dx-\int \mu_{12}^{j}\left(x\right)dx}$$

where $\mu_{1}^{j}\left(x\right)$ and $\mu_{2}^{j}\left(x\right)$ are the membership functions of the *j*-th attribute of Class 1 and Class 2, respectively. $\mu_{12}^{j}\left(x\right)$ is the *j*-th attribute’s membership function of the overlapping area between Class 1 and Class 2, namely the membership function of the compound subset {12}.

Then, a similarity matrix of the *j*-th attribute, $SM_{j}$, is obtained as follows:

(16) $$SM_{j}=\begin{array}{cc} & \begin{array}{cccc} \mu_{1}^{j}\left(x\right) & \mu_{2}^{j}\left(x\right) & \cdots & \mu_{n}^{j}\left(x\right) \end{array}\\ \begin{array}{c} \mu_{1}^{j}\left(x\right)\\ \mu_{2}^{j}\left(x\right)\\ \vdots \\ \mu_{n}^{j}\left(x\right) \end{array} & \left[\begin{array}{cccc} 1 & sim_{12}^{j} & \cdots & sim_{1n}^{j}\\ sim_{21}^{j} & 1 & \cdots & sim_{2n}^{j}\\ \vdots & \vdots & \ddots & \vdots \\ sim_{n1}^{j} & sim_{n2}^{j} & \cdots & 1 \end{array}\right] \end{array}$$

where $sim_{il}^{j}\left(i,l=1,2,\cdots ,n\right)$ is the similarity between class *i* and class *l* in the *j*-th attribute.

Finally, based on the static model of the *j*-th attribute, the reliability of BBA generated from the *j*-th attribute is denoted as follows:

(17) $$R_{j}^{s}=\sum_{i<l}\left(1-sim_{il}^{j}\right)$$

where j=1,2,⋯,k and $sim_{il}^{j}\left(i,l=1,2,\cdots ,n\right)$ is the similarity between class *i* and class *l* in the *j*-th attribute. Obviously, $R_{j}^{s}$ implies that the larger the similarity, the lower the reliability of BBA generated from this attribute model.

**Figure 5:**
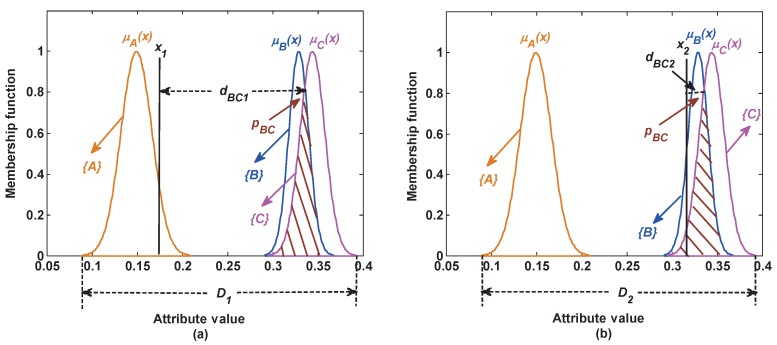
Reliability measure based on the dynamic test samples.

2. The dynamic reliability index based on the test samples:

The static reliability index shows that the overlapping area among classes is larger, the similarity is larger, the possibility of generating the false BBA is larger, then the reliability of the generated BBA is smaller. This index can reflect that the reliability of BBA is affected by some static factors, such as the similarity among classes. However, the reliability of BBA is also affected by the test samples. For example, as shown in Figure 5a, although classes *B* and *C* have a large overlapping area, it is almost impossible that the test sample $x_{1}$ from class *A* is incorrectly classified as class *B* or *C*. In this case, the overlap degree between class *B* and *C* has little negative influence on the reliability of the generated BBA; whereas, in Figure 5b, the test sample $x_{2}$ from class *C* is easily incorrectly classified as class *B*. In this case, the overlap degree between classes *B* and *C* has a large negative influence on the reliability of the generated BBA. As analyzed above, the reliability of BBA is related to the test samples. To reflect this influence, a risk distance *d* is introduced in this section. It is denoted as the distance between the test sample and the overlapping area among attribute models. If the distance is larger, the influence of the overlapping area is smaller, the risk of the incorrect classification is smaller, then the reliability of the generated BBA is larger, and vice versa. The risk distance is produced as follows.

In the attribute j(j=1,2,⋯,k), $p_{12}^{j}$ is the maximum of the intersection between Class 1 and Class 2, namely the vertex of the overlapping area between Class 1 and Class 2. In this section, $p_{12}^{j}$ is taken as the reference point of this overlapping area. Hence, the distance between the test sample $x^{j}$ and the reference point $p_{12}^{j}$ can be calculated to represent the risk distance. This reference point is defined as:

(18) $$p_{12}^{j}=sup\;min\left(\mu_{1}{\left(x\right)}^{j},\mu_{2}{\left(x\right)}^{j}\right).$$

where $\mu_{1}{\left(x\right)}^{j}$ and $\mu_{2}{\left(x\right)}^{j}$ are the Gaussian membership functions of Class 1 and Class 2 in the *j*-th attribute, respectively. Then, a vector $P_{j}$ containing all reference points of the overlapping area among class i(i=1,2,⋯,n) in the *j*-th attribute is obtained as follows:

(19) $$P_{j}=\left[\begin{array}{cccccccc} p_{12}^{j} & \cdots & p_{1n}^{j} & p_{23}^{j} & \cdots & p_{2n}^{j} & \cdots & p_{(n-1)n}^{j} \end{array}\right]$$

In the *j*-th attribute, the risk distance between the test sample $x^{j}$ and the reference points $p_{(n-1)n}^{j}$ of the overlapping area between the classes $\mu_{n-1}^{j}\left(x\right)$ and $\mu_{n}^{j}\left(x\right)$ is formulated as:

(20) $$d_{(n-1)n}^{j}=d\left(x^{j},p_{(n-1)n}^{j}\right)=\frac{|x^{j}-p_{(n-1)n}^{j}|}{D^{j}}$$

where $D^{j}$ represents the maximal interval comprised by all classes. Then, distance vector $D_{j}^{*}$ of the *j*-th attribute is given as follows:

(21) $$D_{j}^{*}=\left[\begin{array}{cccccccc} d_{12}^{j} & \cdots & d_{1n}^{j} & d_{23}^{j} & \cdots & d_{2n}^{j} & \cdots & d_{(n-1)n}^{j} \end{array}\right]$$

Finally, the dynamic reliability index based on the test samples of the *j*-th attribute is denoted as follows:

(22) $$R_{j}^{d}=e^{\sum_{l=2}^{n}d_{(l-1)l}^{j}}$$

where $d_{(l-1)l}^{j}$ is the distance between the test sample $x^{j}$ and the reference points $p_{(l-1)l}^{j}$ of the overlapping area between class $\mu_{l-1}^{j}\left(x\right)$ and $\mu_{l}^{j}\left(x\right)$ in the *j*-th attribute. Obviously, $R_{j}^{d}$ implies that the larger the risk distance, the larger the reliability of BBA generated from this attribute.

3. Comprehensive reliability measure:

Based on the above analysis, a method of measuring the BBA reliability is proposed. This method, which considers the comprehensive reliability based on the attribute model and the test samples, is more reasonable and effective. It is denoted as follows:

(23) $$R_{j}=R_{j}^{s}\times R_{j}^{d}$$

where $R_{j}^{s}$ and $R_{j}^{d}$ are separately the static reliability index and the dynamic reliability index in the *j*-th attribute, which can be gained using Equations (17) and (22), respectively.

Suppose that there are *K* attributes of each class, the reliability of the generated BBA in the *j*-th attribute are normalized as:

(24) $$R_{j}^{*}=\frac{R_{j}}{max(R_{k})}$$

where k=1,2,⋯,K.

According to Section 3.2.1 and Section 3.2.2, both the first component BBA and the second component *R* of reliability-based BBA can be acquired. Finally, the proposed reliability-based BBA (BBA,R) is obtained.

### 3.3. Sensor Data Fusion

In this paper, the reliability-based BBA is first translated into the classical BBA based on the discounting method [16]. After that, Dempster’s combination rule can be used to fuse these BBAs.

Assuming that in the frame of discernment $\Theta =\{\theta_{1},\theta_{2},\cdots ,\theta_{n}\}$, there are *k* reliability-based BBAs $\left(BBA_{j},R_{j}\right),\left(j=1,2,\cdots ,k\right)$. Based on the discounting method, the BBAs generated from sensors are discounted as follows:

(25) $$\left\{\begin{array}{c} m_{j}^{R}\left(A\right)=R_{j}\times m_{j}\left(A\right)\;\;\;\forall A\subset 2^{\Theta }\\ m_{j}^{R}\left(\Theta \right)=R_{j}\times m_{j}\left(\Theta \right)+\left(1-R_{j}\right) \end{array}\right.$$

where $R_{j}$ is the reliability of $BBA_{j}$ of the *j*-th attribute.

Eventually, these reliability-based BBAs are fused using Dempster’s combination rule. The maximum pignistic probability is taken as the decision-making criterion in this paper. Hence, the final mass function is transformed to pignistic probability, and the final decision-making can be done.

## 4. Numerical Example

To evaluate the validity of the reliability-based BBA, several experiments of two datasets and a fault diagnosis are performed in this section.

### 4.1. Experiments on Two Datasets: Five-Fold Cross-Validation

**Figure 6:**
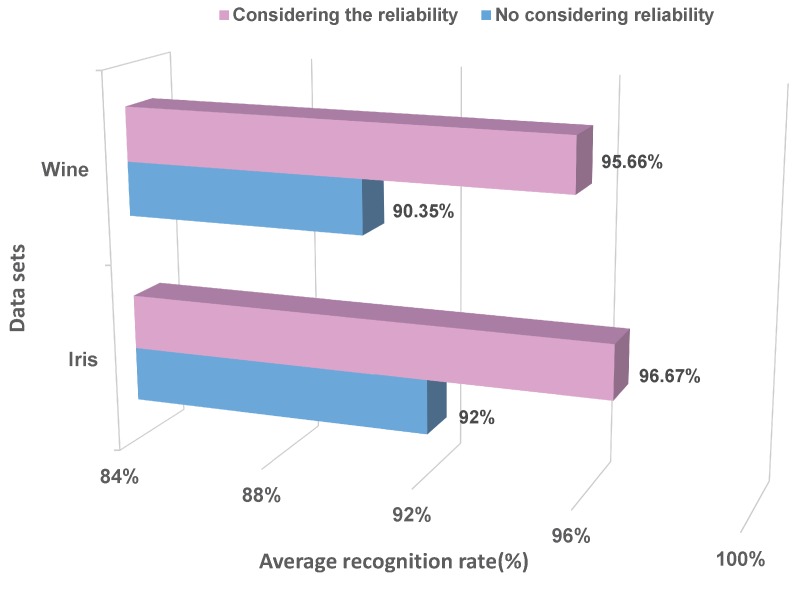
The comparison of considering the reliability or not.

In this section, two kinds of datasets, including Iris and Wine, are selected from the UCI databases (UCI Machine Learning Repository: http://archive.ics.uci.edu/ml/datasets.html.) to evaluate the proposed method. Within these, the Iris dataset contains three classes of 50 samples each, and each class has four attributes, which is the well-known database in pattern recognition. The Wine dataset contains three classes, and each class has 13 attributes. First of all, the proposed method is compared with the method, which uses the same BBA method, but does not consider the reliability of BBA. The comparison results of five-fold cross-validation are shown in Figure 6.

The above results show that after considering the reliability of BBA, the recognition rates of two datasets are all increased to some extent. Namely, the proposed method that measures the reliability of BBA is valid and reasonable.

**Figure 7:**
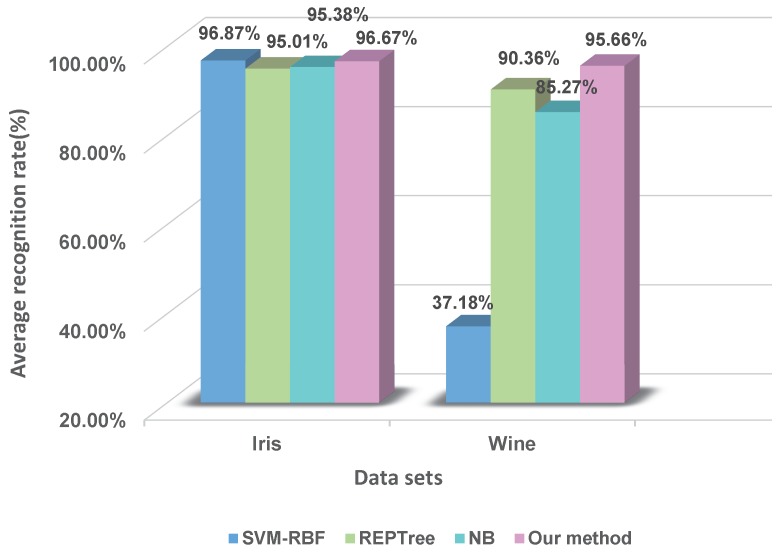
The comparing results between our method and other classifiers.

To further evaluate this method, a comparison experiment between our method and other classifiers is carried out. Here, we consider the following three kinds of classifiers: support vector machine with radial basis function (SVM-RBF), decision tree (REPTree) and naive Bayesian (NB). Within these, SVM and NB are both the top ten data-mining algorithms [51]. REPTree is also a well-known machine-learning algorithm. The comparison results of five-fold cross-validation are shown in Figure 7.

From the above experimental results, it can be found that: in the Iris dataset, the recognition rates of the proposed method and the classical classifiers are greater than 90%, namely they are all effective in this dataset. In the Wine dataset, the recognition rate of our method is 95.66%; NB is 85.27%; REPTree is 90.36%; and SVM-RBF is 37.18%. This shows that the proposed method has competitive performances contrasting with these selected classifiers. What is more, the reliability of BBA is measured at the stage of BBA generation, which is more reasonable.

### 4.2. An Application Example of Fault Diagnosis

To evaluate the validity of this method in engineering applications, a case study of the fault diagnosis of motor rotor is executed. There are three kinds of fault: $F_{1}=\left\{Rotorunbalance\right\}$, $F_{2}=\left\{Rotormisalignment\right\}$ and $F_{3}=\left\{Pedestallooseness\right\}$. Three vibration acceleration sensors and a vibration displacement sensor are placed in different installation positions to collect the vibration signal. Vibration displacement and acceleration vibration frequency amplitudes at the frequencies of 1×, 2× and 3× are taken as the fault feature variables. The relevant data are acquired from the literature [52], which is cited in the Appendix. The method is compared with the method that does not consider the reliability of BBA and other classifiers, respectively. The comparison results of five-fold cross-validation are shown in Table 1.

The above experiments can evaluate the rationality and the effectiveness of the presented method well. The advantages of our method are concluded as follows:

- Based on the idea of the Z-number, an ordered pair (BBA,R) is proposed to represent BBA along with its associated reliability. The first component BBA is a mass function; the second component *R* is a measurement of the reliability of the first component. According to this ordered pair, the reliability of BBA can be measured well at the stage of BBA generation.
- In the process of measuring the reliability of BBA, the information about two things is taken into account. One is the similarity among classes (static information). Another is the risk distance between the test samples and the overlapping area among classes (dynamic information). This makes the results truer and more credible.
- The proposed method is based on a feasible method of measuring the reliability of BBA, which can be replaced with other measure methods for different applications. Namely, this method is flexible and easy to extend in many applications.

## 5. Conclusions

In the multi-sensors data fusion based on evidence theory, how to determine BBA is an open issue. In this paper, a novel method named the reliability-based BBA is proposed. Within this method, first, the models of training samples are built using the Gaussian membership functions. Second, the BBA of every test sample is generated based on the matching degree between the test sample and the attribute model. Then, the reliability of BBA is measured according to both the similarity among classes and the risk distance between the test samples and the overlapping area among classes. Finally, a reliability-based BBA can be generated. Several performed experiments verify that the proposed method is effective. In the future, we will try to apply the proposed method to more practical applications and research a new combination rule for reliability-based BBA.

## Figures and Tables

**Table 1:** The comparing the results of fault diagnosis.

Methods	Classes			Overall Average
	$F_{1}$	$F_{2}$	$F_{3}$	
Not considering reliability	99.00%	95.50%	100%	98.17%
Support vector machine	94.15%	92.86%	100%	95.67%
Decision tree	99.05%	98.68%	99.78%	99.17%
Naive Bayesian	98.05%	96.94%	100%	98.33%
The proposed method	100%	100%	100%	100%

## Appendix A. Experimental Data of Fault Diagnosis

There are three kinds of faults in a motor rotor: F={X,Y,Z}={Rotorunbalance,Rotormisalignment,Pedestallooseness}, which has four fault feature variables. At the same time interval, each fault feature of each fault is continuously observed 40 times, which is taken as a group of observations. A total of five groups is measured in each fault feature of each fault. Hence, i=1,2,3,4 respectively represent four fault feature variables: displacement, acceleration frequency of 1×, 2× and 3×. j=1,2,3,4,5 respectively represent the group numbers of the measured data. Five groups of observations of each feature variables of each fault are shown in Table A1.

**Table A1:** Experimental data of fault diagnosis [52].

Groups	Observations									
**X11**	0.1663	0.1590	0.1568	0.1485	0.1723	0.2006	0.1903	0.1908	0.1986	0.1843
	0.1785	0.1610	0.1579	0.1511	0.1532	0.1647	0.1628	0.1646	0.1634	0.1642
	0.1648	0.1640	0.1674	0.0661	0.1659	0.1650	0.1633	0.1632	0.1604	0.1542
	0.1555	0.1562	0.1540	0.1564	0.1557	0.1542	0.1546	0.1571	0.1537	0.1536
**X12**	0.154	0.1518	0.1537	0.1548	0.1542	0.1538	0.1545	0.1537	0.1571	0.1560
	0.1584	0.1552	0.1586	0.1574	0.1569	0.1565	0.1551	0.1585	0.1585	0.1593
	0.1548	0.1558	0.1547	0.1593	0.1532	0.1632	0.1575	0.159	0.1594	0.1541
	0.165	0.1674	0.1651	0.1604	0.1787	0.1818	0.1820	0.1656	0.1658	0.1644
**X13**	0.1647	0.1647	0.1654	0.1651	0.1656	0.1653	0.1652	0.1652	0.1648	0.1649
	0.1653	0.1650	0.1650	0.1652	0.1653	0.1652	0.1648	0.1647	0.1646	0.1645
	0.1651	0.1652	0.1652	0.1649	0.1650	0.1643	0.1640	0.1639	0.1641	0.1633
	0.1632	0.1629	0.1630	0.1630	0.1634	0.1631	0.1634	0.1629	0.1632	0.1629
**X14**	0.1630	0.1629	0.1627	0.1626	0.1622	0.1624	0.1627	0.1618	0.1614	0.1617
	0.1621	0.1615	0.1618	0.1611	0.1614	0.1610	0.1612	0.1611	0.1616	0.1612
	0.1612	0.1613	0.1623	0.1616	0.1621	0.1613	0.1611	0.1610	0.1610	0.1613
	0.1615	0.1616	0.1618	0.1616	0.1614	0.1612	0.1606	0.1614	0.1619	0.1614
**X15**	0.1609	0.1610	0.1612	0.1615	0.1609	0.1606	0.1604	0.1606	0.1605	0.1601
	0.1604	0.1608	0.1610	0.1603	0.1599	0.1601	0.1602	0.1599	0.1598	0.1598
	0.1598	0.1596	0.1595	0.1593	0.1594	0.1598	0.1596	0.1597	0.1595	0.1593
	0.1598	0.1596	0.1597	0.1595	0.1593	0.1577	0.1580	0.1576	0.1577	0.1579
**X21**	0.1612	0.1620	0.1612	0.1610	0.1385	0.1222	0.1475	0.1306	0.1210	0.1501
	0.1548	0.1577	0.1622	0.1618	0.1621	0.1665	0.1639	0.1652	0.1625	0.1637
	0.1645	0.1645	0.1650	0.1649	0.1650	0.1630	0.1493	0.1533	0.1474	0.1460
	0.1489	0.1499	0.1495	0.1491	0.1489	0.1503	0.1507	0.1505	0.1477	0.1496
**X22**	0.1517	0.1496	0.1504	0.1498	0.1528	0.1519	0.1534	0.1516	0.1555	0.1520
	0.1512	0.1546	0.1538	0.1551	0.1563	0.1536	0.1543	0.1519	0.1514	0.1520
	0.1501	0.1514	0.1483	0.1499	0.1502	0.1550	0.1537	0.1507	0.1557	0.1537
	0.1556	0.1545	0.1529	0.1500	0.1380	0.1343	0.1346	0.1544	0.1458	0.1424
**X23**	0.1464	0.1460	0.1446	0.1448	0.1476	0.1464	0.1434	0.1432	0.1450	0.1420
	0.1448	0.1452	0.1456	0.1462	0.1464	0.1464	0.1444	0.1440	0.1422	0.1442
	0.1470	0.1478	0.1468	0.1482	0.1472	0.1462	0.1478	0.1494	0.1488	0.1496
	0.1480	0.1476	0.1502	0.1496	0.1488	0.1488	0.1484	0.1534	0.1490	0.1486
**X24**	0.1466	0.1460	0.1438	0.1458	0.1488	0.1466	0.1494	0.1502	0.1486	0.1488
	0.1512	0.1490	0.1470	0.1478	0.1484	0.1490	0.1474	0.1456	0.1464	0.1446
	0.3468	0.1484	0.1478	0.1486	0.1470	0.1448	0.1460	0.1458	0.1458	0.1456
	0.1452	0.1470	0.1470	0.1458	0.1450	0.1456	0.1462	0.1442	0.1464	0.1468
**X25**	0.1484	0.1474	0.1488	0.1460	0.1462	0.1464	0.1452	0.1450	0.1438	0.1434
	0.1438	0.1438	0.1436	0.1436	0.1432	0.1412	0.1428	0.1418	0.1422	0.1422
	0.1432	0.1406	0.1420	0.1402	0.1410	0.1418	0.1432	0.1450	0.1418	0.1424
	0.1412	0.1408	0.1412	0.1390	0.1412	0.1398	0.1406	0.1394	0.1392	0.1382
**X31**	0.1221	0.1219	0.1207	0.1215	0.1222	0.1296	0.1235	0.1295	0.1280	0.1233
	0.1218	0.1159	0.1163	0.1195	0.1190	0.1271	0.1247	0.1232	0.1233	0.1237
	0.1210	0.1227	0.1233	0.1222	0.1252	0.1230	0.1049	0.1033	0.0899	0.1003
	0.1044	0.1060	0.1064	0.1042	0.1072	0.1071	0.1070	0.1070	0.1041	0.1049
**X32**	0.1068	0.1063	0.1069	0.1057	0.1091	0.1061	0.1094	0.1067	0.1109	0.1111
	0.1112	0.1096	0.1074	0.1085	0.1109	0.1116	0.1110	0.1113	0.1106	0.1110
	0.1091	0.1080	0.1044	0.1098	0.1084	0.1102	0.1078	0.1087	0.1111	0.1116
	0.1124	0.1128	0.1110	0.1078	0.1101	0.1115	0.1131	0.1108	0.1111	0.1079
**X33**	0.1105	0.1092	0.1074	0.1096	0.1055	0.1076	0.1003	0.1031	0.1040	0.1046
	0.1041	0.1021	0.1041	0.1053	0.1057	0.1038	0.1029	0.1037	0.1012	0.0997
	0.1020	0.1020	0.0990	0.1049	0.1066	0.1065	0.1098	0.1102	0.1076	0.1116
	0.1097	0.1150	0.1120	0.1078	0.1106	0.1075	0.1061	0.1090	0.1098	0.1105
**X34**	0.1105	0.1081	0.1075	0.1059	0.1097	0.1105	0.1086	0.1085	0.1095	0.1084
	0.1093	0.1113	0.1122	0.1139	0.1140	0.1129	0.1119	0.1107	0.1119	0.1137
	0.1128	0.1122	0.1104	0.1129	0.1130	0.1143	0.1132	0.1132	0.1115	0.1111
	0.1123	0.1124	0.1117	0.1120	0.1130	0.1127	0.1158	0.1145	0.1138	0.1144
**X35**	0.1160	0.1137	0.1159	0.1164	0.1158	0.1165	0.1167	0.1160	0.1155	0.1175
	0.1170	0.1175	0.1168	0.1191	0.1190	0.1191	0.1190	0.1211	0.1196	0.1187
	0.1191	0.1202	0.1200	0.1205	0.1194	0.1193	0.1195	0.1180	0.1190	0.1194
	0.1197	0.1194	0.1173	0.1187	0.1169	0.1179	0.1184	0.1197	0.1194	0.1196
**X41**	4.4090	4.3780	4.3430	4.2950	4.2890	4.2890	4.2740	4.1840	4.1820	4.2020
	4.2130	4.2240	4.2220	4.2250	4.2160	4.2220	4.2210	4.2410	4.2200	4.2180
	4.2260	4.2430	4.2390	4.2370	4.2270	4.2300	4.2210	4.2200	4.2430	4.6660
	4.4540	4.4370	4.4380	4.4410	4.4400	4.4350	4.4330	4.4430	4.4460	4.4420
**X42**	4.4480	4.4380	4.4420	4.4320	4.4270	4.4320	4.4220	4.4320	4.4240	4.4270
	4.4590	4.4240	4.4650	4.4180	4.4200	4.4180	4.4190	4.4230	4.4200	4.4460
	4.4210	4.4040	4.4120	4.4000	4.4100	4.4150	4.4070	4.4120	4.3920	4.4020
	4.3930	4.3920	4.3860	4.3890	4.3820	4.3790	4.4120	4.3750	4.3740	4.3790
**X43**	4.3680	4.3840	4.3800	4.3690	4.3840	4.3830	4.3830	4.3820	4.3830	4.3850
	4.3800	4.3800	4.3710	4.3720	4.3740	4.3890	4.3720	4.3670	4.3750	4.3650
	4.3600	4.3570	4.3640	4.3570	4.3550	0.3570	4.3480	4.3470	4.3470	4.3400
	4.3460	4.3360	4.3190	4.3300	4.3480	4.3500	4.3500	4.3460	4.3500	4.3500
**X44**	4.4000	4.3440	4.3410	4.3420	4.3510	4.3450	4.3370	4.3370	4.3340	4.3330
	4.3330	4.3210	4.3250	4.3180	4.3300	4.3100	4.3190	4.3160	4.3160	4.3150
	4.3090	4.3040	4.3060	4.3050	4.3010	4.3000	4.2960	4.2940	4.2860	4.2860
	4.2890	4.2940	4.2900	4.3070	4.2890	4.2800	4.2820	4.2880	4.2810	4.2980
**X45**	4.3000	4.2930	4.2980	4.3303	4.2990	4.2870	4.3030	4.2910	4.2950	4.3050
	4.3020	4.3120	4.3250	4.3090	4.3240	4.3210	4.3240	4.3230	4.3270	4.3290
	4.3230	4.3290	4.3290	4.3350	4.3210	4.3240	4.3270	4.4620	4.4120	4.3740
	4.3960	4.3730	4.3550	4.3540	4.3500	4.3430	4.3470	4.3550	4.3380	4.3310
**Y11**	0.1666	0.1666	0.1670	0.1696	0.1665	0.1671	0.1652	0.1663	0.1656	0.1656
	0.1659	0.1655	0.1640	0.1634	0.1631	0.1618	0.1617	0.1615	0.1597	0.1592
	0.1584	0.1585	0.1575	0.1578	0.1573	0.1567	0.1834	0.1825	0.1827	0.1822
	0.1828	0.1817	0.1820	0.1823	0.1808	0.1818	0.1814	0.1816	0.1808	0.1807
**Y12**	0.1809	0.1794	0.1799	0.1799	0.1788	0.1795	0.1785	0.1785	0.1780	0.1777
	0.1778	0.1777	0.1766	0.1767	0.1761	0.1770	0.1757	0.1765	0.1755	0.1755
	0.1746	0.1757	0.1741	0.1743	0.1741	0.1732	0.1736	0.1723	0.1730	0.1708
	0.1708	0.1702	0.1691	0.1686	0.1683	0.1676	0.1668	0.1670	0.1649	0.1644
**Y13**	0.1637	0.1639	0.1655	0.1641	0.1643	0.1641	0.1625	0.2038	0.2037	0.2033
	0.2014	0.2028	0.2022	0.2026	0.2014	0.2013	0.2007	0.2012	0.1999	0.2012
	0.1998	0.1999	0.1988	0.1992	0.1988	0.1985	0.1977	0.1976	0.1979	0.1981
	0.1966	0.1973	0.1979	0.1984	0.1973	0.1969	0.1963	0.1960	0.1953	0.1941
**Y14**	0.1958	0.1952	0.1954	0.1938	0.1940	0.1956	0.1945	0.1937	0.1954	0.1947
	0.1950	0.1955	0.1947	0.1956	0.1945	0.1932	0.1942	0.1925	0.1924	0.1934
	0.1904	0.1905	0.1909	0.1906	0.1989	0.1898	0.1891	0.1892	0.1886	0.1880
	0.1884	0.1890	0.1874	0.1872	0.1880	0.1855	0.1862	0.1866	0.1849	0.1841
**Y15**	0.1857	0.1844	0.1837	0.1831	0.1835	0.1826	0.1828	0.1834	0.1833	0.1837
	0.1822	0.1829	0.1823	0.1807	0.1833	0.1835	0.1832	0.1828	0.1816	0.1820
	0.1805	0.1808	0.1803	0.1795	0.1785	0.1794	0.1795	0.1788	0.1786	0.1781
	0.1771	0.1775	0.1774	0.1769	0.1780	0.1778	0.1758	0.1740	0.1736	0.1738
**Y21**	0.3111	0.3124	0.3205	0.3268	0.3225	0.3268	0.3305	0.3245	0.3247	0.3245
	0.3300	0.3279	0.3265	0.3221	0.3209	0.3227	0.3196	0.3150	0.3193	0.3182
	0.3148	0.3122	0.3133	0.3107	0.3131	0.3071	0.3412	0.3401	0.3357	0.3466
	0.3422	0.3390	0.3372	0.3364	0.3398	0.3392	0.3384	0.3383	0.3344	0.3394
**Y22**	0.3386	0.3342	0.3364	0.3338	0.3381	0.3388	0.3347	0.3348	0.3321	0.3367
	0.3367	0.3322	0.3300	0.3309	0.3346	0.3341	0.3335	0.3303	0.3320	0.3317
	0.3295	0.3265	0.3299	0.3267	0.3271	0.3253	0.3297	0.3247	0.3243	0.3269
	0.3229	0.3211	0.3171	0.3202	0.3170	0.3125	0.3144	0.3165	0.3079	0.3087
**Y23**	0.3117	0.3095	0.3152	0.3222	0.3171	0.3169	0.3157	0.3480	0.3498	0.3469
	0.3447	0.3476	0.3507	0.3470	0.3403	0.3359	0.3412	0.3399	0.3459	0.3449
	0.3479	0.3422	0.3446	0.3471	0.3467	0.3461	0.3421	0.3413	0.3416	0.3457
	0.3423	0.3439	0.3423	0.3465	0.3405	0.3399	0.3372	0.3387	0.3333	0.3349
**Y24**	0.3419	0.3436	0.3510	0.3392	0.3354	0.3350	0.3500	0.3354	0.3358	0.3349
	0.3385	0.3414	0.3351	0.3394	0.3371	0.3374	0.3370	0.3365	0.3342	0.3389
	0.3386	0.3394	0.3374	0.3355	0.3357	0.3312	0.3274	0.3353	0.3351	0.3325
	0.3305	0.3314	0.3304	0.3238	0.3315	0.3259	0.3253	0.3308	0.3215	0.3233
**Y25**	0.3282	0.3208	0.3211	0.3138	0.3144	0.3199	0.3182	0.3196	0.3205	0.3180
	0.3166	0.3170	0.3181	0.3139	0.3212	0.3254	0.3238	0.3193	0.3204	0.3168
	0.3148	0.3204	0.3146	0.3132	0.3191	0.3164	0.3141	0.3165	0.3137	0.3160
	0.3135	0.3137	0.3188	0.3177	0.3193	0.3239	0.3158	0.3236	0.3291	0.3262
**Y31**	0.2517	0.2634	0.2590	0.2808	0.2869	0.2827	0.2913	0.2909	0.2893	0.2903
	0.2999	0.2961	0.2930	0.3040	0.2971	0.3125	0.2968	0.2979	0.2998	0.3003
	0.3023	0.2986	0.3008	0.3022	0.3017	0.3218	0.2338	0.2414	0.2510	0.2498
	0.2424	0.2451	0.2477	0.2473	0.2494	0.2523	0.2523	0.2496	0.2557	0.2591
**Y32**	0.2485	0.2534	0.2636	0.2670	0.2661	0.2641	0.2581	0.2637	0.2733	0.2735
	0.2644	0.2622	0.2669	0.2713	0.2663	0.2720	0.2753	0.2758	0.2722	0.2755
	0.2710	0.2870	0.2820	0.2770	0.2727	0.2761	0.2812	0.2777	0.2880	0.2919
	0.2882	0.2784	0.2788	0.2792	0.2799	0.2731	0.2717	0.2851	0.2606	0.2696
**Y33**	0.2786	0.2774	0.2921	0.2991	0.2982	0.2974	0.2980	0.2015	0.1872	0.1865
	0.2016	0.1980	0.1982	0.2022	0.2071	0.2020	0.1882	0.1877	0.2065	0.2057
	0.2052	0.2143	0.2135	0.2261	0.2110	0.2077	0.2089	0.2134	0.2161	0.2119
	0.2109	0.2130	0.2180	0.2096	0.2102	0.2152	0.2137	0.2110	0.2113	0.2126
**Y34**	0.2170	0.2130	0.2190	0.2192	0.2112	0.2214	0.2166	0.2137	0.2109	0.2024
	0.2117	0.2102	0.2087	0.2050	0.2149	0.2134	0.2067	0.2140	0.2239	0.2153
	0.2144	0.2103	0.2145	0.2190	0.2250	0.2137	0.2060	0.2153	0.2132	0.2160
	0.2079	0.2047	0.2130	0.2058	0.2174	0.2138	0.2142	0.2138	0.2022	0.2169
**Y35**	0.2206	0.2133	0.2141	0.2031	0.2073	0.2099	0.2066	0.2052	0.2172	0.2131
	0.2140	0.2184	0.2152	0.2099	0.2258	0.2264	0.2273	0.2322	0.2204	0.2248
	0.2242	0.2251	0.2222	0.2317	0.2193	0.2262	0.2255	0.2332	0.2299	0.2289
	0.2305	0.2398	0.2401	0.2306	0.2365	0.2398	0.2439	0.2595	0.2529	0.2557
**Y41**	5.3920	5.3260	5.3080	5.2620	5.2800	5.2460	5.1950	5.2280	5.1840	5.1820
	5.1590	5.1310	5.0980	4.9840	5.0190	4.9340	4.9260	4.9500	4.9690	4.8960
	4.7990	4.8330	4.8220	4.7450	4.7840	4.8260	4.8960	4.8920	4.9120	4.8390
	4.8230	4.7960	4.8000	4.8180	4.8240	4.8310	4.8370	4.8720	4.8410	4.8410
**Y42**	4.8610	4.8220	4.6890	4.7250	4.7070	4.7300	4.6980	4.6810	4.6620	4.7610
	4.7460	4.6870	4.7120	4.7080	4.6910	4.5130	4.4670	4.5120	4.5410	4.3910
	4.4220	4.5130	4.5950	4.5810	4.5420	4.5400	4.5160	4.5220	4.5180	4.5660
	4.5380	4.5450	4.4510	4.4570	4.4810	4.4860	4.4940	4.4690	4.4180	4.4170
**Y43**	4.3700	4.4000	4.3950	4.3840	4.3740	4.3800	4.3310	4.3230	4.3140	4.2870
	4.2300	4.2440	4.2500	4.2200	4.2150	4.2540	4.2100	4.1980	4.2550	4.2210
	4.2110	4.2000	4.1810	4.1790	4.1840	4.1570	4.1440	4.1600	4.1150	4.0940
	4.1230	4.1280	5.2340	5.2320	5.2110	5.2210	5.2280	5.2060	5.1800	5.1890
**Y44**	5.1510	5.1240	5.1230	5.1220	5.0830	5.0600	5.0930	5.0750	5.0490	5.0520
	5.0150	5.0250	5.0750	5.0150	4.9010	4.9300	4.9080	4.8860	4.8780	4.9040
	4.8980	4.8830	4.8510	4.8510	4.8370	4.9340	8.8960	4.8160	4.7640	4.7940
	4.8010	4.7670	4.7450	4.7540	4.7710	4.7560	4.7540	4.7360	4.6780	4.6650
**Y45**	4.6770	4.6610	4.6500	4.6280	4.6440	4.6320	4.6120	4.4620	4.6770	4.6580
	4.6290	4.6220	4.6300	4.6140	4.6260	4.6130	4.5850	4.5690	4.5820	4.5500
	4.5330	4.5520	4.5040	4.4760	4.5660	4.5280	4.5550	4.5230	4.5190	4.5390
	4.5220	4.5210	4.5090	4.4870	4.5270	4.4730	4.4710	4.4900	4.4570	4.4510
**Z11**	0.3207	0.3213	0.3213	0.3235	0.3322	0.3419	0.3434	0.3440	0.3454	0.3461
	0.3474	0.3476	0.3432	0.3468	0.3439	0.3423	0.3440	0.3430	0.3436	0.3420
	0.3416	0.3402	0.3373	0.3403	0.3414	0.3423	0.3420	0.3423	0.3425	0.3379
	0.3379	0.3391	0.3386	0.3355	0.3352	0.3361	0.3333	0.3333	0.3315	0.3347
**Z12**	0.3347	0.3320	0.3323	0.3327	0.3329	0.3287	0.3304	0.3312	0.3285	0.3287
	0.3309	0.3270	0.3274	0.3285	0.3283	0.3305	0.3274	0.3261	0.3264	0.3251
	0.3271	0.3252	0.3275	0.3275	0.3287	0.3270	0.3269	0.3297	0.3266	0.3308
	0.3293	0.3304	0.3323	0.3305	0.3305	0.3330	0.3339	0.3342	0.3312	0.3315
**Z13**	0.3312	0.3301	0.3315	0.3307	0.3315	0.3320	0.3311	0.3327	0.3292	0.3301
	0.3315	0.3289	0.3246	0.3267	0.3295	0.3270	0.3238	0.3264	0.3251	0.3264
	0.3260	0.3247	0.3224	0.3235	0.3249	0.3230	0.3232	0.3273	0.3249	0.3270
	0.3218	0.3244	0.3006	0.3030	0.3041	0.3174	0.3220	0.3196	0.3241	0.3251
**Z14**	0.3263	0.3266	0.3282	0.3270	0.3290	0.3198	0.3237	0.3229	0.3261	0.3238
	0.3259	0.3221	0.3309	0.3271	0.3242	0.3235	0.3240	0.3261	0.3294	0.3287
	0.3267	0.3277	0.3263	0.3262	0.3278	0.3276	0.3271	0.3267	0.3289	0.3270
	0.3266	0.3299	0.3068	0.3148	0.3322	0.3323	0.3320	0.3336	0.3326	0.3322
**Z15**	0.3326	0.3317	0.3301	0.3316	0.3336	0.3280	0.3292	0.3297	0.3283	0.3283
	0.3264	0.3279	0.3275	0.3294	0.3245	0.3268	0.3261	0.3262	0.3253	0.3272
	0.3270	0.3252	0.3284	0.3253	0.3265	0.3277	0.3291	0.3287	0.3256	0.3239
	0.3248	0.3261	0.3252	0.3249	0.3254	0.3290	0.3275	0.3274	0.3274	0.3251
**Z21**	0.2893	0.2863	0.2801	0.2847	0.3271	0.3448	0.3409	0.3346	0.3249	0.3425
	0.3360	0.3368	0.3361	0.3411	0.3434	0.3459	0.3460	0.3481	0.3518	0.3495
	0.3478	0.3477	0.3506	0.3470	0.3470	0.3501	0.3477	0.3561	0.3489	0.3529
	0.3539	0.3544	0.3525	0.3515	0.3560	0.3596	0.3567	0.3616	0.3602	0.3589
**Z22**	0.3541	0.3561	0.3607	0.3636	0.3614	0.3595	0.3586	0.3575	0.3574	0.3563
	0.3601	0.3619	0.3647	0.3599	0.3621	0.3647	0.3557	0.3457	0.3558	0.3509
	0.3525	0.3527	0.3484	0.3452	0.3474	0.3438	0.3500	0.3447	0.3429	0.3508
	0.3397	0.3375	0.3503	0.3421	0.3421	0.3362	0.3328	0.3409	0.3391	0.3364
**Z23**	0.3287	0.3323	0.3313	0.3416	0.3315	0.3352	0.3396	0.3349	0.3402	0.3406
	0.3472	0.3526	0.3439	0.3462	0.3427	0.3492	0.3507	0.3550	0.3456	0.3522
	0.3480	0.3397	0.3474	0.3499	0.3503	0.3365	0.3450	0.3516	0.3506	0.3528
	0.3493	0.3546	0.2995	0.3094	0.2950	0.3479	0.3361	0.3394	0.3484	0.3441
**Z24**	0.3469	0.3380	0.3356	0.3378	0.3385	0.3338	0.3396	0.3345	0.3363	0.3426
	0.3333	0.3298	0.3335	0.3339	0.3397	0.3349	0.3357	0.3361	0.3401	0.3382
	0.3379	0.3356	0.3309	0.3333	0.3328	0.3330	0.3412	0.3334	0.3264	0.3297
	0.3302	0.3318	0.2961	0.3143	0.3616	0.3506	0.3463	0.3446	0.3412	0.3393
**Z25**	0.3454	0.3396	0.3453	0.3455	0.3517	0.3426	0.3590	0.3516	0.3481	0.3502
	0.3440	0.3428	0.3455	0.3404	0.3518	0.3517	0.3389	0.3481	0.3382	0.3530
	0.3471	0.3566	0.3554	0.3539	0.3576	0.3536	0.3480	0.3568	0.3567	0.3524
	0.3587	0.3578	0.3535	0.3602	0.3565	0.3490	0.3532	0.3541	0.3507	0.3467
**Z31**	0.1810	0.1864	0.1803	0.1829	0.1605	0.1441	0.1436	0.1412	0.1414	0.1476
	0.1502	0.1477	0.1507	0.1469	0.1490	0.1512	0.1461	0.1497	0.1511	0.1488
	0.1486	0.1480	0.1493	0.1451	0.1520	0.1537	0.1498	0.1478	0.1471	0.1496
	0.1467	0.1443	0.1446	0.1420	0.1454	0.1365	0.1347	0.1373	0.1380	0.1446
**Z32**	0.1434	0.1380	0.1413	0.1412	0.1452	0.1444	0.1396	0.1364	0.1400	0.1424
	0.1408	0.1419	0.1415	0.1393	0.1472	0.1452	0.1387	0.1383	0.1267	0.1326
	0.1326	0.1398	0.1283	0.1291	0.1296	0.1282	0.1314	0.1235	0.1283	0.1179
	0.1206	0.1285	0.1365	0.1290	0.1290	0.1345	0.1191	0.1275	0.1290	0.1187
**Z33**	0.1252	0.1210	0.1268	0.1339	0.1333	0.1359	0.1309	0.1362	0.1315	0.1399
	0.1387	0.1369	0.1326	0.1381	0.1308	0.1301	0.1322	0.1302	0.1260	0.1241
	0.1266	0.1210	0.1298	0.1264	0.1232	0.1250	0.1313	0.1284	0.1257	0.1281
	0.1321	0.1350	0.1665	0.1695	0.1692	0.1386	0.1352	0.1422	0.1409	0.1332
**Z34**	0.1387	0.1343	0.1349	0.1335	0.1289	0.1300	0.1282	0.1263	0.1258	0.1331
	0.1268	0.1291	0.1353	0.1295	0.1304	0.1279	0.1345	0.1329	0.1329	0.1294
	0.1398	0.1386	0.1318	0.1278	0.1371	0.1317	0.1357	0.1361	0.1370	0.1416
	0.1291	0.1350	0.1368	0.1535	0.1340	0.1304	0.1312	0.1331	0.1276	0.1302
**Z35**	0.1232	0.1340	0.1316	0.1299	0.1375	0.1238	0.1344	0.1229	0.1331	0.1324
	0.1297	0.1297	0.1233	0.1286	0.1314	0.1334	0.1259	0.1362	0.1151	0.1279
	0.1256	0.1287	0.1323	0.1216	0.1263	0.1296	0.1241	0.1274	0.1252	0.1310
	0.1276	0.1314	0.1328	0.1284	0.1284	0.1339	0.1346	0.1360	0.1356	0.1359
**Z41**	9.7920	9.8090	9.8090	9.8130	9.8190	9.8730	9.7850	9.8220	9.7880	9.7530
	9.8170	9.7530	9.7060	9.7480	9.7840	9.7210	9.7330	9.7910	9.9090	9.9510
	9.9670	9.9340	9.8760	9.9070	9.9470	9.8780	9.9150	9.9200	9.9090	9.9220
	9.8440	9.8740	9.8000	9.8700	9.8970	9.8670	9.8760	9.8830	9.9370	9.9330
**Z42**	9.9070	9.8530	9.8510	9.8690	9.8250	9.8630	9.8610	9.8440	9.8500	9.7980
	9.8300	9.8250	9.8370	9.8890	9.8350	9.8030	9.7550	9.7960	9.7760	9.7730
	9.7270	9.6260	9.6430	9.6620	9.6920	9.6800	9.6990	9.3850	9.7020	9.7160
	9.7420	9.6530	9.7390	9.7830	9.7030	9.7460	9.7360	9.8000	9.7490	9.7840
**Z43**	9.7060	9.7540	9.7830	9.7500	9.7290	9.7900	9.7790	9.7370	9.7640	9.6970
	9.6850	9.7260	9.6830	9.6880	9.7230	9.7360	9.6930	9.7560	9.7500	9.7880
	9.7050	9.7660	9.7710	9.8240	9.8610	9.8290	9.8020	9.8550	9.7600	9.8230
	9.8610	9.8200	9.8420	9.8370	9.8340	9.8750	9.9040	9.8570	9.8000	9.8650
**Z44**	9.8190	9.8400	9.8350	9.7756	9.8520	9.8900	9.9230	9.8810	9.9580	9.9290
	9.9320	9.6500	9.9680	9.9220	9.8580	9.9460	9.8760	9.9400	9.8370	9.7400
	9.8990	9.9440	9.9570	10.0360	9.8960	9.9550	10.0230	10.0170	9.9950	9.7420
	9.6220	9.7320	9.7280	9.9780	10.1120	10.0350	9.9930	9.6710	9.5720	9.6780
**Z45**	9.7530	9.7570	9.7510	9.8330	9.7730	9.7980	9.8460	9.8440	9.8750	9.8690
	9.8300	9.6950	9.6930	9.6990	9.6540	9.6880	9.5790	9.6610	9.9250	9.8580
	9.6240	9.6830	9.8540	9.6300	9.5890	9.6450	9.7990	9.8260	9.9420	9.9150
	9.9150	9.7980	9.9240	9.8970	9.8820	9.8090	9.7990	9.8150	9.8580	9.8380
